# γ-Herpesvirus Load as Surrogate Marker of Early Death in HIV-1 Lymphoma Patients Submitted to High Dose Chemotherapy and Autologous Peripheral Blood Stem Cell Transplantation

**DOI:** 10.1371/journal.pone.0116887

**Published:** 2015-02-10

**Authors:** Chiara Pratesi, Stefania Zanussi, Rosamaria Tedeschi, Maria Teresa Bortolin, Renato Talamini, Maurizio Rupolo, Chiara Scaini, Giancarlo Basaglia, Matteo Di Maso, Mario Mazzucato, Ernesto Zanet, Umberto Tirelli, Mariagrazia Michieli, Antonino Carbone, Paolo De Paoli

**Affiliations:** 1 Microbiology, Immunology and Virology Unit, CRO National Cancer Institute, IRCCS, Aviano, Pordenone, Italy; 2 Epidemiology and Biostatistics Unit, CRO National Cancer Institute, IRCCS, Aviano, Pordenone, Italy; 3 Cellular Therapy and High-Dose Chemotherapy Unit, CRO National Cancer Institute, IRCCS, Aviano, Pordenone, Italy; 4 Stem Cell Collection and Processing Unit, CRO National Cancer Institute, IRCCS, Aviano, Pordenone, Italy; 5 Division of Medical Oncology A, CRO National Cancer Institute, IRCCS, Aviano, Pordenone, Italy; 6 Department of Pathology, CRO National Cancer Institute, IRCCS, Aviano, Pordenone, Italy; 7 Scientific Directorate; CRO National Cancer Institute, IRCCS, Aviano, Pordenone, Italy; University of British Columbia, CANADA

## Abstract

Autologous stem cell transplantation (ASCT) is a feasible procedure for human immunodeficiency virus-1 (HIV-1) lymphoma patients, whose underlying disease and intrinsic HIV-1- and ASCT-associated immunodeficiency might increase the risk for γ-herpesvirus load persistence and/or reactivation. We evaluated this hypothesis by investigating the levels of Epstein-Barr virus (EBV)- and Kaposi sarcoma-associated herpesvirus (KSHV)-DNA levels in the peripheral blood of 22 HIV-1-associated lymphoma patients during ASCT, highlighting their relationship with γ-herpesvirus lymphoma status, immunological parameters, and clinical events. EBV-DNA was detected in the pre-treatment plasma and peripheral blood mononuclear cells (PBMCs) of 12 (median 12135 copies/mL) and 18 patients (median 417 copies/10^6^ PBMCs), respectively; the values in the two compartments were correlated (r = 0.77, p = 0.0001). Only EBV-positive lymphomas showed detectable levels of plasma EBV-DNA. After debulking chemotherapy, plasma EBV-DNA was associated with lymphoma chemosensitivity (p = 0.03) and a significant higher mortality risk by multivariate Cox analysis adjusted for EBV-lymphoma status (HR, 10.46, 95% CI, 1.11–98.32, p = 0.04). After infusion, EBV-DNA was detectable in five EBV-positive lymphoma patients who died within six months. KSHV-DNA load was positive in only one patient, who died from primary effusion lymphoma. Fluctuations in levels of KSHV-DNA reflected the patient’s therapy and evolution of his underlying lymphoma. Other γ-herpesvirus-associated malignancies, such as multicentric Castleman disease and Kaposi sarcoma, or end-organ complications after salvage treatment were not found. Overall, these findings suggest a prognostic and predictive value of EBV-DNA and KSHV-DNA, the monitoring of which could be a simple, complementary tool for the management of γ-herpesvirus-positive lymphomas in HIV-1 patients submitted to ASCT.

## Introduction

The human γ-herpesviruses, Epstein Barr virus (EBV) and Kaposi sarcoma-associated herpesvirus (KSHV) are DNA tumor viruses that establish lifelong latent infections in B lymphocytes. The frequency of their reactivation is associated with altered cell-mediated immune functions, such as the one occurring in HIV-1 infection, where EBV and KSHV concur in the development of AIDS related disorders [1,2]. Also, HIV-1 negative patients who are iatrogenically immunosuppressed following allogenic transplantation [3–5] are at risk of developing severe lymphoproliferative and end-organ diseases, mediated by γ-herpesviruses reactivation [6]. Although rare, cases of γ-herpesviruses related neoplastic and non-neoplastic complications have been observed in immunocompetent patients who received autologous stem cell transplantation (ASCT) for haematological diseases [7–11].

Thanks to combination Antiretroviral Therapy (cART), significantly reducing HIV-1 replication and improving reconstitution of antiviral immunity [12], also HIV-1-associated lymphoma patients can be admitted to salvage ASCT-based treatments [13–18]. However, even when immune reconstitution after ASCT occurs with dynamics overlapping the seronegative counterpart, skewing of T cell receptor repertoire [19] and lower levels of CD4^+^ T lymphocytes are observed during follow-up. Moreover, an increased incidence of opportunistic infections in the first 100 days post-transplantation has been reported [20].

Due to the intrinsic HIV-1 related immune senescence [21,22] and to the further immunosuppression induced by previous cytotoxic treatments, it cannot be excluded that HIV-1 patients undergoing salvage treatment with ASCT might be at high risk for post-transplant γ-herpesviruses reactivation and related clinical events. In addition, since 30% to 90% of AIDS-related systemic lymphomas are associated with γ-herpesviruses [23], their replication may indicate the underlying lymphoma [24–27] that significantly affects prognosis of HIV-1 auto-transplanted patients [14].

Measuring γ-herpesvirus-DNA load in peripheral blood is now a common laboratory tool for monitoring γ-herpesvirus infections [28], and several studies have highlighted its value as a prognostic and predictive parameter in AIDS-related lymphomas [24,25,27,29,30]. In this retrospective analysis, concerning one of the largest mono-institutional series of HIV-1-associated relapse/refractoring lymphoma patients who underwent ASCT, peripheral blood EBV-DNA and KSHV-DNA loads were evaluated in order to assess their level and the frequency of γ-herpesviruses reactivation, as well as their predictive and prognostic value during the ASCT procedure and follow-up.

## Material and Methods

### Patients and Samples

This retrospective, observational, virological and immunological study included all consecutive, refractory, or relapsed HIV-1-associated lymphoma patients, who attended our Institute from 2001 to 2007, received salvage treatment with high dose chemotherapy (HDC) plus ASCT, and showed at least 2-month follow-up after transplantation with available samples. The Ethics Committee of the National Cancer Institute-IRCCS Aviano approved this study, and a written informed consent was obtained in accordance with the Declaration of Helsinki. All the patients’ information was de-identified prior to analysis. The ASCT procedure and pharmacological treatments have been described elsewhere [20,31]. In brief, all the patients were submitted to intensive, conventional, second-line debulking chemotherapy (DCT) according to the type of lymphoma and based on ESHAP (etoposide, cytarabine, cisplatinum, methylprednisone), DHAOx (dexamethasone, high-dose cytarabine, and oxaliplatinum) or MINE schemes (mesna, iphosphamide, mitoxantrone, etoposide, prednisolone). DCT containing Rituximab was administered to 10 out of 11 patients having a CD20-positive non Hodgkin lymphoma (NHL). Autografts were collected after enhancement of CD34^+^ stem cell mobilization with granulocyte colony stimulating factor. The second phase of the treatment consisted of a conditioning regimen with HDC BEAM (carmustine, etoposide, cytarabine, melphalan). Two days after the end of the HDC treatment, the cryopreserved autograft was infused. cART was administered to all patients during the ASCT procedure. Anti-cytomegalovirus therapy and/or prophylaxis was administered to 17/22 patients from the beginning of the conditioning regimen until neutrophil recovery.

Virological and immunological parameters were evaluated before DCT (i.e., at lymphoma relapse), post-DCT (i.e., immediately before HDC plus autograft infusion), as well as at months 0.5 (aplastic period), 1, 3, 6 and 12 after autograft infusion, namely until recovery of at least baseline CD4^+^ T cell counts was attained [20]. Moreover, one case was assessed at year 3 in conjunction with relapse occurrence. Due to medical history and sampling gaps, plasma and/or peripheral blood mononuclear cell (PBMC) samples were not always available for all the patients. Death from any cause and relapse/occurrence of γ-herpesvirus-associated malignances were considered as events. Moreover, any clinical event other than death or relapse was assessed in correspondence to a γ-herpesvirus quantitative determination.

Information on outcomes was obtained through verification of patients’ vital status and lymphoma relapse from the date of enrollment to the date of the last visit. The median follow-up of the patients after autograft infusion was 50 months (range 2–109 months).

### *In situ* Hybridization and Immunohistochemical Analysis

Tissue samples from 19 out of 22 patients at diagnosis were available for pathological revision and γ-herpesvirus lymphoma status determination. All lymphoma cases were classified according to WHO classification system [32]. EBV status was determined by EBV-Encoded RNAs (EBER) in situ hybridization, and immunohistochemical analysis for latent membrane protein (LMP-1) expression on formalin-fixed, paraffin-embedded tissue sections. KSHV lymphoma status was assessed in available cases with potential KSHV-associated histology (i.e., one plasmablastic lymphoma, one extracavitary/solid variant of primary effusion lymphoma) or with positive KSHV viremia (one diffuse large B cell lymphoma (DLBCL) of immunoblastic type). KSHV status was determined by immunohistochemical analysis for latent LANA-1 (KSHV ORF-73) expression on formalin-fixed, paraffin-embedded tissue sections.

### Viral Load Quantification

Cryo-preserved sample aliquots of 200 μl plasma and of about 2 × 10^6^ PBMCs were processed for DNA extraction with the QIAamp blood Mini kit (Qiagen), according to the manufacturer’s instructions. EBV-DNA and KSHV-DNA were measured by real time TaqMan PCR by using the ABI PRISM 7900 HT Sequence Detection System (Applied Biosystems), as previously described [29–33]. An EBV-DNA standard curve was generated by 10-fold serial dilutions of Namalwa genomic DNA, ranging from 5 to 5000 copies per reaction. The analytic validation assays showed that the sensitivity of the method allowed the quantification of 1, 2.5, and 5 copies of target DNA per reaction in 33%, 75%, and 90% of cases, respectively. The lack of inhibitory material in each sample was confirmed in an additional reaction seeded with a known amount of Namalwa genomic DNA. By using this method, EBV-DNA was always undetectable in plasma and detectable in the PBMCs of 35% of healthy donors [34].

Since the KSHV-DNA and EBV-DNA detection systems were comparable, the number of KSHV genomes in each sample was calculated from the EBV-DNA standard curve [33]. The lack of inhibitory material in each sample submitted to KSHV quantification was confirmed in an additional reaction seeded with a known amount of DNA extracted from a KSHV-infected BCBL1 lymphoma cell line. Results were expressed as copies of γ-herpesvirus genomes per milliliter of plasma or per 10^6^ PBMCs. Normalization of the γ-herpesviruses-DNA copies per 10^6^ PBMCs in each peripheral blood DNA sample was obtained by real time TaqMan PCR on human β-globin gene, as already reported [34]. The β-globin standard curve was obtained by 10-fold serial dilutions of human genomic DNA, ranging from 5 to 5000 copies per reaction. For statistical purposes, a viral load of zero was assigned to samples in which γ-herpesvirus-DNA was undetectable.

The absolute lymphocyte subset counts were evaluated by a single platform whole blood lysing technique and with the EPICS XL flow cytometer (Beckman-Coulter), as described elsewhere [35]. Plasma HIV-RNA levels were assessed by Quantiplex HIV bDNA assay (3.0 Siemens; detection limit 50 copies/mL). For statistical purposes, a viral load of 49 copies/mL was assigned to samples with HIV-RNA below the threshold. The HIV-DNA level was measured by real time PCR with primers and TaqMan probe detecting a 121-bp DNA fragment in the viral LTR region [31].

### Definitions and Statistical Analyses

The Spearman’s rank correlation coefficient was used to analyze the correlation between the virological and immunological covariates before and post-DCT. Comparisons of matched and unmatched data were made using Wilcoxon signed rank and Wilcoxon rank sum tests, respectively. McNemar’s test was used to assess the difference between pre- and post-DCT frequency of EBV-DNA positivity in matched plasma (N = 20) and PBMCs (N = 16) samples. Fisher’s exact test was used to compare the proportions of patients with EBV-positive lymphoma to subjects with EBV-negative lymphoma by Rituximab treatment and to evaluate the association between EBV-DNA positivity post-DCT and the response to DCT assessed by radiological examination. Response to DCT treatment was evaluated according to International Workshop criteria [36] as complete response (CR), unconfirmed CR (uCR), partial response (PR), stable disease and progressive disease. Patients with a decrease in measurable disease ranging from 75% to 100% (CR + uCR) were considered as objective responders, subjects with disease reduction <50% or stable disease and progressive disease were categorized together as not responders.

The risk of mortality was assessed from the date before DCT until death for any cause after autograft infusion. The risk of lymphoma relapse was measured from the date before DCT to lymphoma relapse after autograft infusion defined by radiological examination and immunohistological or cytological evaluation of tissue biopsies or needle aspirates. Data were censored at the time of last available follow-up. Univariate Cox proportional hazards regression analysis [37] was used to estimate the association between the outcome (mortality and lymphoma relapse) and risk factors including age, histology, Ann Arbor stage, EBV lymphoma status, Rituximab treatment during DCT, CD4^+^ T cell counts, CD4/CD8 T cell ratio, CD19^+^ B cell counts, HIV-DNA load and EBV-DNA load. The predictive values of the EBV-DNA load on outcome were examined in a multivariate Cox model adjusted for EBV lymphoma status. The survival probability curve for plasma EBV-DNA levels was assessed by the Kaplan-Meier survivor functions, and the differences between variable groups were evaluated by the χ^2_1_^log-rank K test [38]. Analyses were performed with SAS System software, version 8.2 (SAS Institute Inc., Cary, NC, USA, 1999–2001). Results were considered statistically significant at a 2-tailed p-value ≤ 0.05.

## Results

### Patients

Twenty-two consecutive HIV-1 patients with a refractory or relapsed lymphoma who underwent ASCT and met the selection criteria were included in this study. Their clinical, virological, and immunological characteristics before DCT were summarized in Table 1 (data for each patient are reported in S1 and S2 Table). The median age was 42 years (range: 29–67) and the male/female ratio was 20:2. The most frequent histological subtype was DLBCL NHL (59.1%), followed by Hodgkin lymphoma (HL) (22.7%). Approximately 96% of the patients had lymphomas at 3 and 4 Ann Arbor Stages. Four out five HLs and eight out of fourteen NHLs were classified as EBV-positive tumors. Only the extracavitary/solid variant of primary effusion lymphoma (PEL) showed KSHV ORF-73 and EBER positivity. None of the patients had clinical or histological signs of Kaposi sarcoma (KS) or multicentric Castleman disease (MCD), neither at diagnosis nor at relapse. The median CD4^+^ T cell count was 189 cells/μL (range: 13–630 cells/μL), and 26.3% of the patients had a severe immune depression (CD4^+^ T cell count < 100 cells/μL). Detectable HIV-RNA levels were found in 7/20 patients, with significant post-DCT reduction, as previously described [20]; only in three patients HIV-RNA remained detectable. Within one year after autograft infusion, 13 patients (59.1%) were alive, 6 (27.3%) died due to lymphoma’s relapse, and 3 (13.6%) died due to infection.

**Table 1:** Demographic, clinical, histological, virological and immunological characteristics of 22 HIV-1 lymphoma patients before debulking chemotherapy.

**Characteristics**	**Values**
Age (median years, range)	42 (29–67)
Gender (n, %)	
Female	2 (9.1)
Male	20 (90.9)
**Lymphoma status (n, %)**	
Relapsed lymphoma	17 (77.3)
Refractory lymphoma	5 (22.7)
Ann Arbor Stage (n, %)	
2	1 (4.5)
3	9 (41.0)
4	12 (54.5)
Lymphoma histotype (n, %)	
DLBCL (5 EBV-positive lymphoma/11 total tested)	13 (59.1)
Immunoblastic	5 (22.7)
Centroblastic	4 (18.2)
Anaplastic	1 (4.5)
NOS	3 (13.6)
Plasmablastic (1 EBV-positive and KSHV-negative lymphoma/1 total tested)	2 (9.1)
Extracavitary/solid variant PEL (EBV- and KSHV-positive lymphoma)	1 (4.5)
ALCL T (EBV-positive lymphoma)	1 (4.5)
Classic HL (4 EBV-positive lymphoma/5 total tested)	5 (22.7)
EBV lymphoma tissue status (n, %)	
Positive	12 (54.6)
Negative	7 (31.8)
Unknown	3 (13.6)
Plasma EBV-DNA copies/mL (median, range)	135 (0–105400)
Cell-associated EBV-DNA copies/10^6^ PBMCs (median, range)	417 (0–33223)
HIV-RNA copies/mL (median, range)	49 (49–305426)
Cell-associated HIV-DNA copies/10^6^ PBMCs (median, range)	117 (2–479)
CD4 cells/μl (median, range)	189 (13–630)
CD4/CD8 (median, range)	0.24 (0.06–0.85)
CD19 cells/μl (median, range)	70 (0–419)
CD3^−^56^+^ cells/μl (median, range)	56 (3–999)

Plasma and cell-associated KSHV-DNA were detected in two DLBCL immunoblastic lymphomas (one KSHV-negative in lymphoma tissue; one unknown) and in one extracavitary/solid variant of PEL (KSHV-positive in lymphoma tissue). DLBCL diffuse large cell lymphoma; PEL, primary effusion lymphoma; NOS, not otherwise specified; ALCL, anaplastic large cell lymphoma; HL, Hodgkin’s lymphoma; EBV, Epstein Barr virus; PBMCs, peripheral blood mononuclear cells.

### EBV-DNA Load before DCT

Before DCT, the plasma EBV-DNA was detected in 12/21 patients (57.1%) (overall median: 135 copies/mL; range: 0–105400 copies/mL). EBV-DNA was detected in PBMCs of 18/20 (90.0%) patients (overall median: 417 copies/10^6^ PBMCs; range: 0–33223 copies/10^6^ PBMCs). All the patients with an EBV-positive lymphoma had always-detectable EBV-DNA in plasma (median: 1180 copies/mL; range: 68–105400 copies/mL) and in PBMCs (median: 892 copies/10^6^ PBMCs; range: 123–15498 copies/10^6^ PBMCs). All the patients with EBV-negative lymphoma had always-undetectable plasma EBV-DNA, whereas 4/6 (66.7%) showed detectable EBV-DNA in PBMCs (median: 72 copies/10^6^ PBMCs; range: 11–299 copies/10^6^ PBMCs; p = 0.006 vs. EBV-positive lymphoma patients).

Correlations between EBV-DNA levels and virological and immunological parameters at the time of lymphoma relapse were reported in Table 2. A significant strong correlation was observed between EBV-DNA levels in plasma and in PBMCs. Plasma EBV-DNA was negatively correlated with CD4/CD8 T cell ratio, CD19^+^ B, and with CD3^−^56^+^ NK cell counts. In the cellular compartment, EBV-DNA levels correlated negatively with CD3^−^56^+^ NK cell counts. No significant association between HIV-RNA or HIV-DNA levels and EBV-DNA load was observed, either in plasma or in PBMCs.

**Table 2:** Spearman’s rank correlation coefficients between EBV-DNA levels in plasma or PBMCs compartment and virological or immunological parameters before debulking chemotherapy.

**Plasma EBV-DNA**	**Coefficient (r)**	**p-value**	**Cell-associated EBV-DNA**	**Coefficient (r)**	**p-value**
EBV-DNA in PBMCs	0.77	0.0001			
HIV-RNA	−0.08	0.75	HIV-RNA	0.25	0.32
HIV-DNA	−0.07	0.88	HIV-DNA	0.16	0.52
CD4/CD8	−0.47	0.04	CD4/CD8	−0.44	0.07
CD19 #	−0.49	0.03	CD19 #	−0.24	0.34
CD3^−^56^+^ #	−0.67	0.002	CD3^−^56^+^ #	−0.61	0.008

EBV, Epstein Barr Virus; PBMCs, peripheral blood mononuclear cells, #, cells/μl

### EBV-DNA Load Post-DCT

As compared to before, a lower frequency of EBV-DNA positivity in both plasma and PBMCs was observed post-DCT (5/21 patients, 23.8%; 9/18 patients, 50.0%, respectively). Analysis on matched data confirmed the significant reduction in the proportion of EBV-DNA detected in plasma and PBMCs (p = 0.01 and p = 0.04, respectively). In addition, EBV-DNA levels post-DCT were significantly reduced in both plasma (median: 0 copies/mL; range: 0–1600 copies/mL; p = 0.004 vs. pre-DCT) and PBMCs (median: 6 copies/10^6^ PBMCs; range: 0–1070 copies/10^6^ PBMCs; p = 0.01 vs. pre-DCT). As at the time of lymphoma relapse, the post-DCT plasma EBV-DNA load was detectable only in patients with EBV-positive lymphoma tissue. Of note, the significant decrease in cell-associated EBV-DNA loads between pre- and post-DCT concerned only the subset of Rituximab treated patients (pre-DCT median: 229 copies/10^6^ PBMCs; pre-DCT range: 0–33223 copies/10^6^ PBMCs; post-DCT median: 0 copies/10^6^ PBMCs; post-DCT range: 0–0 copies/10^6^ PBMCs; p = 0.02). EBV-positive lymphomas showed higher residual cell-associated EBV-DNA levels (median: 154 copies/10^6^ PBMCs; range: 0–1024 copies/10^6^ PBMCs) as compared to EBV-negative lymphomas (median: 6 copies/10^6^ PBMCs; range: 0–197 copies/10^6^ PBMCs; p = 0.04), and a lower frequency of them were treated with Rituximab (16.7% EBV-positive lymphomas vs. 85.7% EBV-negative lymphomas, p = 0.006).

**Figure 1:**
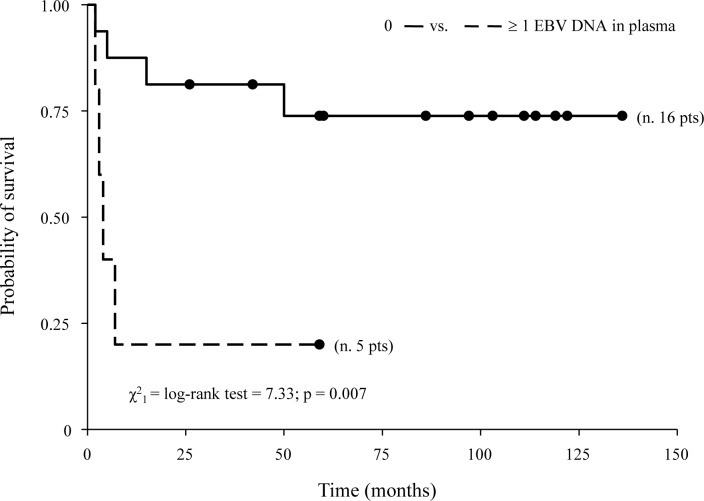
Kaplan-Meier estimates of overall survival by plasma EBV-DNA post-DCT in HIV-1-associated lymphoma patients. DCT, debulking chemotherapy.

Post-DCT, the negative correlation between plasma EBV-DNA and CD4/CD8 T cell ratio was maintained (r = −0.45, p = 0.04). A positive significant correlation between EBV-DNA in PBMCs and CD19^+^ B cell counts emerged (r = 0.69, p = 0.002). A persistent EBV-DNA in plasma was associated with the absence of an objective response to DCT (p = 0.03). The values of some demographic, clinical, and laboratory parameters in predicting the risk of mortality and lymphoma relapse were calculated (Table 3). In univariate Cox regression analysis, only post-DCT EBV-DNA positivity in plasma or PBMCs were associated with a significantly higher mortality risk (HR: 5.88, 95% CI: 1.40–24.66; p = 0.02 and HR: 5.52, 95% CI: 1.08–28.16; p = 0.04, respectively) and with an increased risk, although not statistically significant, of developing lymphoma relapse during follow-up post-autograft infusion (HR: 4.41, 95% CI: 0.94–20.67; p = 0.06; HR: 4.52, 95% CI: 0.85–24.10; p = 0.08, respectively). Given the significant prognostic value of EBV-DNA load on mortality and the concordance between EBV-DNA and EBV lymphoma status before DCT, multivariate Cox analysis adjusted for EBV lymphoma status was performed. The results indicated that plasma EBV-DNA remained the only prognostic variable for mortality (HR: 10.46, 95% CI: 1.11–98.32; p = 0.04). Survival analyses using the Kaplan-Meier method further supported that patients with detectable post-DCT plasma EBV-DNA had poorer survival than did patients with undetectable post-DCT plasma EBV-DNA (20% vs. 81% at month 24; p = 0.007) (Fig. 1).

**Table 3:** Hazard ratio (HR) and corresponding 95% confidence interval (CI) for mortality and lymphoma relapse evaluated by demographic, clinical, histological, virological and immunological parameters.

	**N (%)**	**Mortality**			**Lymphoma Relapse**		
		**HR**	**95%CI**	**p-Value**	**HR**	**95%CI**	**p-Value**
Age (years)							
≤42	11 (50.0)	1 ^†^			1 ^†^		
>42	11 (50.0)	1.21	(0.32–4.51)	0.78	2.46	(0.48–12.72)	0.28
Lymphoma histology							
High grade NHL	17 (77.3)	1 ^†^			1 ^†^		
HL	5 (22.7)	1.07	(0.22–5.15)	0.94	0.63	(0.08–5.29)	0.67
Stage (Ann Arbor)							
2–3	10 (45.5)	1 ^†^			1 ^†^		
4	12 (54.5)	0.90	(0.24–3.37)	0.88	0.88	(0.20–3.96)	0.87
Rituximab							
yes	10 (45.5)	1 ^†^			1 ^†^		
no	12 (54.5)	2.21	(0.53–8.55)	0.29	2.56	(0.49–13.36)	0.26
CD4 post-DCT^‡^(cells/μL)							
< 200	12 (57.1)	1 ^†^			1 ^†^		
≥ 200	9 (42.9)	0.72	(0.17–3.04)	0.66	1.77	(0.40–7.93)	0.45
CD4/CD8 post-DCT^‡^							
≤ 0.28	11 (52.4)	1 ^†^			1 ^†^		
> 0.28	10 (47.6)	1.09	(0.27–4.39)	0.90	1.55	(0.35–6.94)	0.57
CD19 post-DCT^‡^(cells/μL)							
≤ 1	11 (52.4)	1 ^†^			1 ^†^		
> 1	10 (47.6)	2.6	(0.62–10.95)	0.19	2.02	(0.45–9.04)	0.36
Cell-associated HIV-DNA post-DCT^‡^(copies/10^6^ PBMCs)							
< 70	9 (47.4)	1 ^†^			1 ^†^		
≥ 70	10 (52.6)	1.51	(0.36–6.38)	0.57	5.42	(0.65–45.21)	0.12
EBV lymphoma tissue status^‡^							
Negative	7 (36.8)	1 ^†^			1 ^†^		
ositive	12 (63.2)	1.04	(0.25–4.37)	0.96	0.60	(0.12–2.96)	0.53
Plasma EBV-DNA post-DCT^‡^(copies/mL)							
0	16 (76.2)	1 ^†^			1 ^†^		
≥ 1	5 (23.8)	5.88	(1.40–24.66)	0.02	4.41	(0.94–20.67)	0.06
Cell-associated EBV-DNA post-DCT^‡^(copies/10^6^ PBMCs)							
0	9 (50.0)	1 ^†^			1 ^†^		
≥ 1	9 (50.0)	5.52	(1.08–28.16)	0.04	4.52	(0.85–24.10)	0.08

^†^, reference category

^‡^, the sum does not add up to the total because of missing values

DCT, debulking chemotherapy; NHL, non Hodgkin’s lymphoma; HL, Hodgkin’s lymphoma; PBMCs, peripheral blood mononuclear cells.

### EBV-DNA Load after Autograft Infusion

During follow-up after autograft infusion, three patients died due to fatal infections within the first six months after transplantation and two of them, who had an EBV-positive lymphoma, showed EBV-DNA in plasma and PBMCs at the aplastic period, immediately before developing a life-threatening CMV infection (plasma EBV-DNA: 95 and 57 copies/mL; cell-associated EBV-DNA: 1063 and 2540 copies/10^6^ PBMCs).

Lymphoma relapse occurred in 7/22 patients (31.8%), and EBV lymphoma status was known for 6 of them. Three had an EBV-positive lymphoma and, concomitantly, presented detectable EBV-DNA in both compartments (plasma EBV-DNA: 33160, 2573, and 13410 copies/mL; PBMCs EBV-DNA: 37561, 1313 and 187 copies/10^6^ PBMCs). They died for disease progression within four months after autograft infusion. Three relapsed patients had an EBV-negative lymphoma and showed undetectable plasma EBV viremia before and close to the relapse. Two died 13 and 14 months thereafter, and one was still alive at the last follow-up. For the remaining relapsing patient, EBV lymphoma status was unknown, and EBV viremia could not be assessed.

For the remaining 12 patients still alive after transplantation, evidence for serious γ-herpesvirus-associated end-organ diseases was not found when γ-herpesvirus viremia was assessed. New γ-herpesvirus-associated malignances, such as MCD or KS, were not clinically and/or histologically diagnosed until the last visit. In the 63 plasma samples analyzed until year one after transplantation, EBV-DNA was always negative, except for samples from two patients, who showed a sporadic increase in plasma EBV viremia at months 0.5 (324 copies/mL) and 12 (1000 copies/mL), respectively. The first observed viral rise was associated with a persistent fever after recovery from a sepsis; the second one occurred together with appearance of lymphadenopathy and with suspension of antiviral therapy with Famciclovir for an extended cutaneous herpes zoster. Cell-associated EBV-DNA was detected with increasing frequency from month 0.5 to 12 after autograft infusion (4/12 patients at month 0.5, 11/12 patients at month 12; p = 0.01). At year one, the median level of EBV-DNA in PBMCs was 316 copies/10^6^ PBMCs (range: 0 −2083 copies/10^6^ PBMCs), it did not correlate with the B cell counts (r = 0.07, p = 0.83), but, as observed post-DCT, it was inversely correlated with CD4/CD8 T cell ratio (r = −0.74, p = 0.006). Post-autograft infusion immunological and virological data for each patient are reported in S3 Table.

### KSHV-DNA Load during ASCT Procedure

Before DCT, the KSHV-DNA load was detected in plasma samples of 3/16 patients (18.8%) (55, 5936, 57765 copies/mL, in patient #8, #18, #7, respectively); the last two subjects also showed detectable KSHV-DNA in PBMCs (199025 and 14 copies/10^6^PBMCs in patients #18 and #7, respectively). Patients #7 and #8 had an immunoblastic lymphoma, patient #18 was diagnosed with an extracavitary/solid variant of PEL. Tissues for KSHV lymphoma status assessment were available for patients #8 and #18, who showed a KSHV-negative and KSHV-positive tumor, respectively.

Post-DCT, the plasma KSHV load in patients #8 and #7 became undetectable, while in patient #18 a 83% decrease was observed. All three patients showed negative KSHV-DNA in PBMCs.

**Figure 2:**
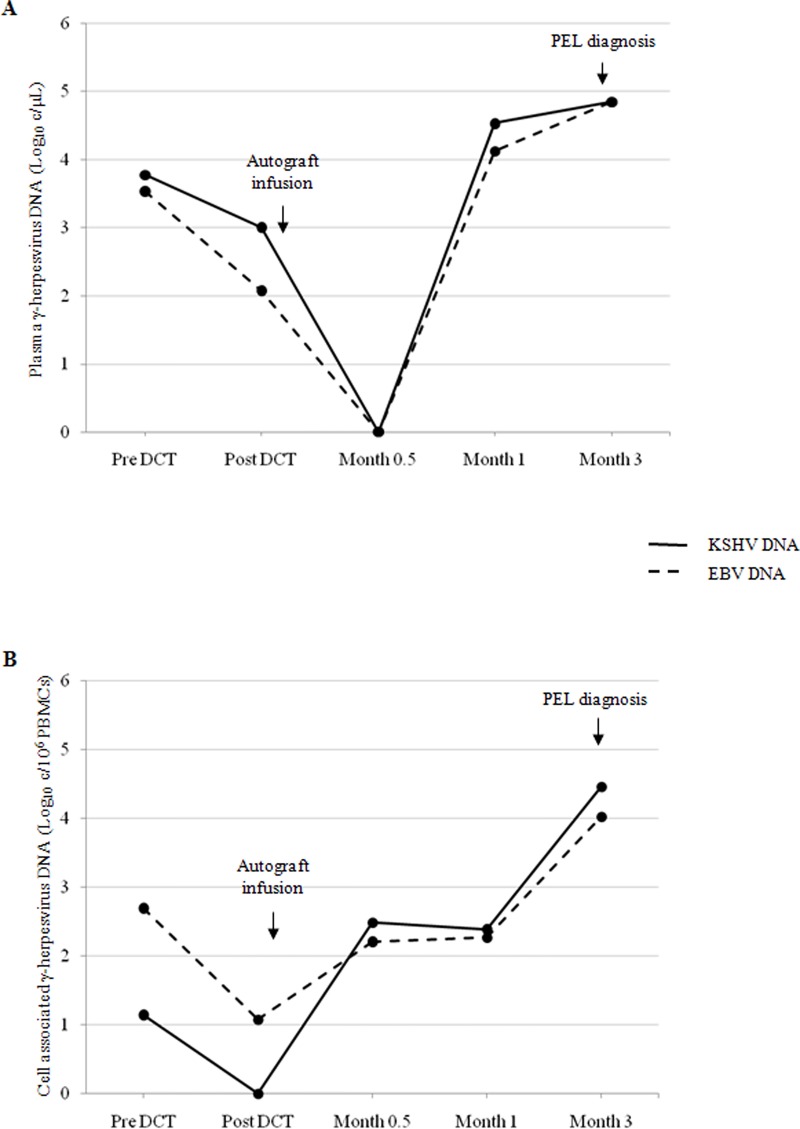
EBV-DNA (dashed line) and KSHV-DNA (solid line) load dynamics in plasma (A) and in PBMCs (B) during pre- and post- transplantation in patient #18. DCT, debulking chemotherapy.

After autograft infusion, the KSHV-DNA and EBV-DNA loads were detectable and consistently increased with the same dynamics during three months after transplantation in one patient (#18) (Fig. 2). At month 3, evolution of the initially diagnosed extracavitary/solid variant of PEL to classic PEL was documented, and the patient died 30 days later. Of note, a sharp rise in the plasma KSHV-DNA and EBV-DNA load between month 0.5 to 1 preceded that one in PBMCs. In this patient, the histological revision of lymphoma tissue at diagnosis and relapse after transplantation revealed the co-expression of KSHV ORF-73 and EBER in the tumor cells.

## Discussion and Conclusions

Several studies have highlighted the association of γ-herpesviruses with AIDS-related systemic lymphomas [23, 26], evidencing the important contribution of γ-herpesvirus infection to lymphoma genesis in the context of heavy immune deficiency. Moreover, in AIDS-related lymphoma patients undergoing ASCT, the immune system is severely compromised by the HIV-1 infection, the tumor itself, prior CT cytotoxic effects, and by ablative conditioning finalized to stem cell infusion. For this reason as well as the severity of the underlying disease, these patients could be at high risk of relapse or of developing new γ-herpesvirus-related neoplastic and non-neoplastic diseases. To the best of our knowledge, this is the first study evaluating EBV-DNA and KHSV-DNA viremia in one of the largest mono-institutional cohorts of HIV-1-associated lymphoma patients submitted to ASCT in order to assess the frequency and the levels of γ-herpesviruses reactivation and their prognostic and predictive value during the ASCT procedure and follow-up.

In this cohort, we found a high percentage of EBV-positive lymphoma cases, in agreement with literature highlighting a role of EBV in lymphoma development, in concert with immune suppression and, presumably, other oncogenic factors [39,40]. At the time of lymphoma relapse, plasma EBV-DNA was absent in patients with EBV-negative lymphoma, as reported for healthy donors [34], while it was always detectable in those with an EBV-positive lymphoma. On the contrary, cell-associated EBV-DNA was found in all EBV-positive patients and in a high proportion of the EBV-negative lymphoma patients. It is worth noting that the EBV-DNA load in both compartments was significantly associated with compromised adaptive and innate immunity. In particular, the inverse correlation between NK cells and EBV-DNA load supports the role of this immunological population in controlling EBV-associated tumor genesis, as evidenced by recent data in murine tumor models [41]. Overall, these results highlighted that EBV-DNA levels could mirror both the presence of an EBV-positive lymphoma and/or the lack of immunological control of active EBV replication, possibly fostering the genesis/progression of the lymphoma, as suggested in pre-clinical studies [42,43]. These data evidence the challenge in the interpretation of this marker, as a consequence of the dual nature of the γ-herpesvirus viremia, i.e., naked tumor-derived viral DNA or virional tumor/benign replication-derived DNA. At present, available molecular techniques that can distinguish among these species are not conclusive and not applicable in the setting of routine monitoring [44–46]. Nevertheless, our study highlighted a complete agreement between EBV lymphoma status and EBV-DNA load only in the plasma compartment, suggesting that most of the plasma EBV-DNA could be tumor-derived and, when compared with cell-associated EBV-DNA, could be more specific in indicating the presence of the lymphoma.

A link between plasma EBV viremia and tumor burden emerged also post-DCT, where the reduced but persistent plasma EBV-DNA load was present only in EBV-associated lymphomas and was related to poor DCT response assessed by radiological examination. In addition, in EBV-positive lymphoma patients, the residual plasma EBV-DNA load significantly predicted poor survival following transplantation, hence resembling the association between lymphoma chemosensitivity and survival after salvage treatment in a similar series of HIV-1 patients [14]. Concerning the EBV-DNA in PBMCs, our results indicate that the purging of the cell-associated EBV-DNA load by debulking treatment was complete in patients treated with DCT plus Rituximab, confirming that Rituximab was more targeted and effective in destroying the B cell compartment. Since among EBV-positive lymphoma patients only three were CD20-positive, the high cell-associated EBV-DNA levels in these subjects could be ascribed to the absence of Rituximab treatment. As already reported by other authors [47–49], overall these findings confirmed that, compared with γ-herpesvirus load in PBMCs, γ-herpesvirus-DNA in plasma is more specific in indicating residual disease and could be useful in prognosis evaluation of EBV-positive tumours.

An estimation of the relationship between KSHV-DNA levels and immune parameters, radiological response to DCT or survival factors was prevented by the low frequency of KSHV-DNA peripheral blood positivity in this as in other series of AIDS-related lymphomas [27,29]. One patient with diagnosis and relapse of extracavitary/solid variant of PEL, co-expressing EBV and KSHV in the lymphomatous cells, showed always-positive plasma KSHV-DNA and EBV-DNA before and after DCT. Moreover, a sharp rise of plasma KSHV-DNA and EBV-DNA load preceded that one in PBMCs and the onset of a classic PEL after transplantation. In the first month after autograft infusion, we observed plasma and cell-associated γ-herpesvirus viremia though this patient was in a context of a seriously compromised immune system with complete absence of peripheral blood B cells. Considering the evolution of the disease in this patient, it is not excludible that γ-herpesvirus load in both compartments represents the lymphoma load rather than immunedepression-induced γ-herpesvirus reactivation. These observations highlight that quantitative KSHV-DNA determination in peripheral blood might be a simple parameter useful in selecting patients who need KSHV-DNA monitoring, especially in light of cases of KSHV reactivation described in HIV-negative patients after transplantation [10,11,50].

Evidence on the potential utility of the γ-herpesvirus viremia, in particular for EBV-DNA, has also been shown after autograft infusion. In fact, the EBV-DNA load in both compartments increased not only just before occurrence of life-threatening opportunistic infections, but also in a considerable proportion (43%) of relapses. All these patients had EBV-positive lymphomas. In addition, they had a shorter follow-up post-relapse than did the aviremic and EBV-negative lymphoma relapsing patients, mirroring evidences supporting a worse prognosis of EBV-positive than EBV-negative lymphomas [51–53].

In the living subjects with complete remission after transplantation, plasma EBV-DNA was mainly negative. By contrast, a progressive increase in the frequency of EBV-DNA positivity in PBMCs was observed and occurred without development of EBV-associated clinical symptoms and relationship with reconstituted B cell numbers. This finding could be the result of viral reactivation consequent to low immunosurveillance, as suggested by the negative association between the CD4/CD8 T cell ratio and EBV-DNA in PBMCs. A definitive interpretation of EBV viremia in PBMCs would benefit from the definition of threshold values.

Presently, no guidelines on a threshold value for EBV-DNA load with clear clinical significance in lymphoproliferations are available due to lack of international consensus on the best calibrator, specimen type, or unit of reporting [54,55]. Furthermore, the chemotherapy regimen is an important influencing factor on tumor-associated EBV-DNA levels, but a useful clinical cut-off indicating residual disease has still not been established. It follows that reliance on a viral load threshold *per se* to predict γ-herpesvirus-associated disease jeopardizes early detection of lymphoma, mostly in previously chemo-treated patients. Our study supports previous findings emphasizing plasma γ-herpesvirus-DNA load as a prognostic and predictive parameter in lymphomas both associated and not associated with HIV-1 [24–27,29,30,47,48], and it suggests that plasma residual γ-herpesvirus load might be a convenient candidate surrogate marker of residual disease [49]. To improve the predictive value of cellular EBV-DNA positivity, EBV-DNA measurements should be included within a prognostic score model combining B cell counts, T cell immunosuppression, EBV specific T cell reactivity [56] and the context of the patients’ chemotherapeutic plan.

Despite the weaknesses, such as the retrospective nature of the investigation, the small number of patients, the lack of a protocol schedule envisaging frequent sample collection, and the impossibility of computing an EBV-DNA reference threshold useful in results interpretation, some observations can be made from this study. Indeed, this is the first one describing the peripheral γ-herpesvirus viremia in one of the largest mono-institutional cohorts of relatively homogeneous relapse/refractoring HIV-1-associated lymphoma patients submitted to ASCT. It was found that salvage treatment does not expose the patients to develop new γ-herpesvirus-associated malignances or end-organ diseases. The evidences also indicated that plasma EBV-DNA positivity is associated with EBV-positive lymphomas, deep immune deficiency, tumor chemosensitivity, and early mortality after transplantation. Finally, the γ-herpesvirus-DNA load could be a useful inexpensive marker for clinical monitoring of γ-herpesvirus-positive HIV-1-associated lymphoma patients submitted to ASCT and could aid decision-makers in defining treatment options.

## Supporting Information

S1 Table Baseline demographic and clinical characteristics of 22 HIV-1 lymphoma patients. (DOC)

S2 Table Immunological and virological parameters before autograft infusion (pre- and post-debulking chemotherapy). (DOC)

S3 Table Immunological and virological parameters in alive patients with complete remission during follow-up after autograft infusion. (DOC)
