# Supplementary material for: γ-Herpesvirus Load as Surrogate Marker of Early Death in HIV-1 Lymphoma Patients Submitted to High Dose Chemotherapy and Autologous Peripheral Blood Stem Cell Transplantation
Source: PLoS One. 2015 Feb 10;10(2):e0116887. doi: 10.1371/journal.pone.0116887 (PMC4323102; doi:10.1371/journal.pone.0116887)
Supplement: S1 Table — (DOC) [file pone.0116887.s001.doc]

| **PTS** | **Sex** | **Age** | **Lymphoma histology** | **EBV Lymphoma tissue status** | **Ann Arbor Stage** | **Rituximab treatment** | **Post-debulking chemotherapy response** | **Lymphoma relapse after autograft infusion** | **Time elapsed between baseline and last visit (yrs)** | **Vital status at last follow-up** |
| --- | --- | --- | --- | --- | --- | --- | --- | --- | --- | --- |
|  |  |  |  |  |  |  |  |  |  |  |
| 1# | F | ≤42 | HG NHL | Negative | 3 | Yes | R | No | 12.0 | Alive |
| 2# | M | ≤42 | HL | Positive | 2 | No | R | No | 10.2 | Alive |
| 3# | M | ≤42 | HG NHL | Positive | 3 | No | R | No | 10.5 | Alive |
| 4# | M | >42 | HL | Positive | 4 | No | R | No | 10.8 | Alive |
| 5# | M | ≤42 | HG NHL | Unknown | 3 | Yes | R | No | 5.3 | Alive |
| 6# | M | ≤42 | HG NHL | Positive | 4 | Yes | R | No | 6.9 | Alive |
| 7# | M | ≤42 | HG NHL | Unknown | 4 | Yes | R | No | 7.6 | Alive |
| 8# | M | ≤42 | HG NHL | Positive | 4 | No | NR | Yes | 0.8 | Dead |
| 9# | M | >42 | HG NHL | Positive | 3 | No | R | No | 8.9 | Alive |
| 10# | M | >42 | HG NHL | Positive | 4 | Yes | R | No | 5.4 | Alive |
| 11# | M | >42 | HG NHL | Negative | 3 | Yes | R | No | 0.6 | Dead |
| 12# | M | ≤42 | HG NHL | Negative | 4 | Yes | R | No | 8.6 | Alive |
| 13# | M | ≤42 | HL | Positive | 4 | No | NR | No | 1.4 | Dead |
| 14# | M | ≤42 | HG NHL | Positive | 3 | No | NR | Yes | 0.4 | Dead |
| 15# | M | >42 | HL | Negative | 3 | No | NR | Yes | 2.5 | Alive |
| 16# | M | >42 | HG NHL | Negative | 4 | Yes | R | Yes | 4.5 | Dead |
| 17# | M | >42 | HG NHL | Negative | 3 | Yes | R | Yes | 1.6 | Dead |
| 18# | M | >42 | HG NHL | Positive | 4 | No | R | Yes | 0.7 | Dead |
| 19# | M | >42 | HG NHL | Unknown | 4 | No | R | Yes | 0.7 | Dead |
| 20# | M | ≤42 | HL | Positive | 3 | No | R | No | 0.6 | Dead |
| 21# | M | >42 | HG NHL | Negative | 4 | Yes | R | No | 3.9 | Alive |
| 22# | F | >42 | HG NHL | Positive | 4 | No | R | No | 5.7 | Alive |

Table S1. Baseline demographic and clinical characteristics of 22 HIV-1 lymphoma patients

Abbreviations: M, male; F, female; HG NHL, High grade Non Hodgkin Lymphoma; HL, Hodgkin Lymphoma; R, objective response; NR, no objective response
