# Supplementary material for: γ-Herpesvirus Load as Surrogate Marker of Early Death in HIV-1 Lymphoma Patients Submitted to High Dose Chemotherapy and Autologous Peripheral Blood Stem Cell Transplantation
Source: PLoS One. 2015 Feb 10;10(2):e0116887. doi: 10.1371/journal.pone.0116887 (PMC4323102; doi:10.1371/journal.pone.0116887)
Supplement: S2 Table — (DOC) [file pone.0116887.s002.doc]

|  | Pre-debulking chemotherapy | | | | | | | | Post-debulking chemotherapy | | | | | |
| --- | --- | --- | --- | --- | --- | --- | --- | --- | --- | --- | --- | --- | --- | --- |
| PTS | CD4 cells/μL | CD4/CD8 | CD19 cells/μL | CD3-CD56+ cells/μL | plasma EBV-DNA copies/mL | cell-associated EBV-DNA copies/10^6^PBMCs | Plasma  KSHV-DNA copies/mL | cell-associated KSHV-DNA copies/10^6^PBMCs | CD4 cells/μL | CD4/CD8 | CD19 cells/μL | CD3-CD56+ cells/μL | plasma EBV-DNA copies/mL | cell associated EBV-DNA copies/10^6^PBMCs |
| 1# | 460 | 0.48 | 419 | 56 | 0 |  | 0 |  | 173 | 0.33 | 1 | 203 | 0 | 0 |
| 2# | 172 | 0.23 | 70 | 166 | 795 | 892 | 0 | 0 | 186 | 0.37 | 8 | 140 | 0 |  |
| 3# | 189 | 0.24 | 129 | 18 | 38562 | 1632 |  |  | 141 | 0.21 | 5 | 247 | 0 |  |
| 4# | 191 | 0.85 | 178 | 51 | 931 | 254 | 0 | 0 | 86 | 0.41 | 10 | 32 | 0 |  |
| 5# | 211 | 0.37 | 218 | 110 | 0 | 332 | 0 | 0 | 398 | 0.59 | 0 | 35 | 0 | 0 |
| 6# | 212 | 0.23 | 175 | 54 | 2346 | 15498 | 0 | 0 | 256 | 0.16 | 0 | 50 | 0 | 0 |
| 7# | 172 | 0.17 | 0 | 7 | 96940 | 33223 | 57765 | 199025 | 327 | 0.28 | 0 | 27 | 0 | 0 |
| 8# | 152 | 0.13 | 37 | 72 | 5110 | 11415 | 55 | 0 | 401 | 0.11 | 4 | 80 | 1172 | 1024 |
| 9# | 293 | 0.17 | 273 | 529 | 68 | 283 |  |  | 251 | 0.24 | 174 | 186 | 0 | 213 |
| 10# | 13 | 0.06 | 11 | 51 | 105400 | 763 | 0 | 0 | 82 | 0.14 | 0 | 45 | 1600 | 0 |
| 11# | 53 | 0.10 | 48 | 79 | 0 | 71 | 0 | 0 |  |  |  |  |  |  |
| 12# |  |  |  |  | 0 | 0 |  |  | 68 | 0.65 | 0 | 13 | 0 | 0 |
| 13# | 76 | 0.13 | 33 | 6 | 135 | 1149 | 0 | 0 | 23 | 0.04 | 1 | 40 | 1077 | 479 |
| 14# |  |  |  |  |  |  |  |  | 99 | 0.41 | 9 | 96 | 1200 | 154 |
| 15# | 630 | 0.69 | 37 | 115 | 0 | 73 | 0 | 0 | 443 | 1.22 | 0 | 99 | 0 | 197 |
| 16# | 369 | 0.35 | 129 | 348 | 0 | 229 |  |  | 179 | 0.33 | 0 | 24 | 0 | 0 |
| 17# | 340 | 0.31 | 87 | 999 | 0 | 0 | 0 | 0 | 274 | 0.29 | 0 | 134 | 0 | 0 |
| 18# | 95 | 0.19 | 1 | 51 | 3445 | 501 | 5936 | 14 | 148 | 0.17 | 74 | 38 | 119 | 12 |
| 19# |  |  |  |  | 0 | 755 | 0 | 0 | 294 | 0.21 | 9 | 99 | 0 | 1070 |
| 20# | 287 | 0.59 | 18 | 53 | 152 | 123 | 0 | 0 | 115 | 0.29 | 4 | 116 | 0 | 280 |
| 21# | 150 | 0.32 | 94 | 99 | 0 | 11 | 0 | 0 | 81 | 0.19 | 0 | 34 | 0 | 0 |
| 22# | 45 | 0.24 | 19 | 3 | 1180 | 2300 |  |  | 203 | 0.27 | 191 | 33 | 0 | 40 |

Table S2. Immunological and virological parameters before autograft infusion (pre- and post-debulking chemotherapy)
