# Supplementary material for: γ-Herpesvirus Load as Surrogate Marker of Early Death in HIV-1 Lymphoma Patients Submitted to High Dose Chemotherapy and Autologous Peripheral Blood Stem Cell Transplantation
Source: PLoS One. 2015 Feb 10;10(2):e0116887. doi: 10.1371/journal.pone.0116887 (PMC4323102; doi:10.1371/journal.pone.0116887)
Supplement: S3 Table — (DOC) [file pone.0116887.s003.doc]

|  | Month 0.5 post-autograft infusion | | | | Month 1 post-autograft infusion | | | | Month 3 post-autograft infusion | | | | Month 6 post-autograft infusion | | |  | Month 12 post-autograft infusion | | | |
| --- | --- | --- | --- | --- | --- | --- | --- | --- | --- | --- | --- | --- | --- | --- | --- | --- | --- | --- | --- | --- |
|  |  |  |  |  |  |  |  |  |  |  |  |  |  |  |  |  |  |  |  |  |
| PTS | CD19 cells/μL | CD4/CD8 | plasma  EBV-DNA copies/mL | cell-associated  EBV-DNA copies/10^6^PBMCs | CD19 cells/μL | CD4/CD8 | plasma  EBV-DNA copies/mL | cell-associated  EBV-DNA copies/10^6^PBMCs | CD19 cells/μL | CD4/CD8 | plasma  EBV-DNA  copies/mL | cell-associated  EBV-DNA copies/10^6^PBMCs | CD19 cells/μL | CD4/CD8 | plasma  EBV-DNA copies/mL | cell-associated  EBV-DNA copies/10^6^PBMCs | CD19 cells/μL | CD4/CD8 | plasma EBV-DNA copies/mL | cell-associated  EBV-DNA copies/10^6^PBMCs |
| 1# | 1 | 1.05 | 0 | 0 | 261 | 0.40 | 0 | 0 | 277 | 0.41 | 0 | 0 | 541 | 0.64 | 0 | 65 | 634 | 1.04 | 0 | 116 |
| 2# | 2 | 0.60 | 324 | 605 | 11 | 0.06 | 0 | 307 | 112 | 0.11 | 0 | 1148 | 105 | 0.18 | 0 | 0 | 148 | 0.17 | 0 | 353 |
| 3# | 0 | 0.58 | 0 | 0 | 2 | 0.31 | 0 | 37 | 183 | 0.15 | 0 | 164 | 339 | 0.20 | 0 | 0 | 570 | 0.29 | 0 | 743 |
| 4# | 2 | 0.33 | 0 | 10 | 50 | 0.33 | 0 | 0 | 132 | 0.35 | 0 | 220 | 128 | 0.36 | 0 | 208 | 283 | 0.42 | 0 | 147 |
| 5# | 1 | 1.14 | 0 | 0 | 0 | 0.18 | 0 |  | 253 | 0.17 | 0 | 0 | 186 | 0.23 | 0 | 0 | 359 | 0.15 | 0 | 337 |
| 6# | 0 | 0.49 | 0 | 0 | 1 | 0.35 | 0 | 0 | 2 | 0.09 | 0 | 0 | 59 | 0.13 | 0 | 680 | 545 | 0.15 | 0 | 1867 |
| 7# | 0 | 0.04 | 0 | 0 | 2 | 0.08 | 0 | 0 | 1 | 0.11 | 0 | 0 |  |  |  |  | 264 | 0.29 | 0 | 19 |
| 9# | 2 | 0.51 | 0 | 4676 |  |  |  |  | 57 | 0.08 | 0 | 981 | 265 | 0.07 | 0 | 109 | 606 | 0.11 | 1000 | 2083 |
| 10# | 0 | 0.38 | 0 | 0 | 0 | 0.01 | 0 | 0 | 0 | 0.06 | 0 | 0 | 25 | 0.05 | 0 | 14 | 70 | 0.05 | 0 | 443 |
| 12# | 0 | 0.18 | 0 | 0 |  |  |  |  | 97 | 0.29 | 0 | 0 | 321 | 0.68 | 0 | 0 | 564 | 0.75 | 0 | 0 |
| 21# | 0 | 0.37 | 0 | 0 | 1 | 0.08 | 0 | 0 | 1 | 0.10 | 0 | 0 | 113 | 0.12 | 0 | 0 | 268 | 0.11 | 0 | 295 |
| 22# | 2 | 0.19 | 0 | 76 |  |  |  |  | 535 | 0.10 | 0 | 560 |  |  |  |  | 479 | 0.61 | 0 | 270 |

Table S3. Immunological and virological parameters in alive patients with complete remission during follow-up after autograft infusion
